# The weakness of weak ties for novel information diffusion

**DOI:** 10.1007/s41109-017-0034-3

**Published:** 2017-06-19

**Authors:** Jennifer M. Larson

**Affiliations:** New York University, Department of Politics, 19 W. 4th St., New York, 10012 NY USA

**Keywords:** Diffusion, Weak ties, Capacity, Random ties, Diversity

## Abstract

Weak ties are thought to facilitate the diffusion of information through social networks because of their tendency to span otherwise distant subgroups. However, this logic assumes that weak relationships have the same capacity to transmit information as those that are strong. I argue that weak ties, especially the kind that span subgroups, are often also lower-capacity. Due to a lack of trust, an unwillingness to share benefits, or a limited ability to understand one another, an individual is less likely to share novel information across these ties. In standard models of diffusion imported from epidemiology, even reduced-capacity links would still aid diffusion. However, accounting for reduced capacity in a new model of diffusion that captures realistic features of information sharing in human groups, I demonstrate that hesitation to share across weak links substantially impedes overall diffusion. Moreover, I show that the addition of weak ties to a social network can strictly reduce the extent and speed of information diffusion. Increasing density by adding weak ties can make diffusion strictly worse by crowding out the use of higher-capacity ties. I present the results of simulated information diffusion on both hypothetical networks generated to possess varying levels of density and homophily, as well as on real social networks in two Ugandan villages shown to be responsible for face-to-face information sharing.

## Introduction

Interpersonal relationships can vary in intensity; those that are weak are thought to serve valuable roles in social networks. Because weak ties tend to bridge otherwise distant subgroups, their presence spreads information originating in one subgroup to other subgroups, improving diffusion (Granovetter 1973). This logic treats “weakness” as a property of the interpersonal relationship with no bearing on the tie’s information-transmitting capacity. Implicitly, the logic assumes that so long as a weak tie has non-zero capacity, its presence will be beneficial for the reach of information.

This view of information dissemination draws on the standard approach to diffusion on a network, which uses insights from epidemiology to explain how information might spread from person to person in a social network (Banerjee et al. 2013; Jackson and Rogers 2007; Newman 2002; Siegel 2009; Valente 1996; Young 2009). According to these approaches, nodes “infected” with an idea are “contagious”; network neighbors of the infected are exposed and hence susceptible to the infection, with variants accounting for the consequences of exposure to multiple sources (Centola and Macy 2007; Centola 2013), variation in motivation (Granovetter 1978; Chwe 2000), the cumulative effect of repeated exposures (Dodds and Watts 2004, 2005), and homophily with respect to susceptibility (Chiang 2007).

While the epidemiological approach has offered valuable insights, ties in a social network function quite differently for the spread of information than ties in a contact network function for the spread of a disease. In the case of a contact network, a tie by definition makes an alter susceptible to the disease of the ego. In the case of a social network, a tie does not *by definition* spread information to an alter. A tie indicates a social relationship. Whether or not this social relationship results in an ego passing information to an alter depends on a variety of factors: whether the two happen to encounter each other while the information is salient, whether they are together for long enough for the information to come up, whether the ego thinks the information is relevant to the alter, whether the ego is willing to share with the particular alter, and so on. Moreover, these factors are likely correlated with the strength of the social relationship between the ego and the alter.

Considering the type of information that is often the subject of diffusion studies, an ego may have good reason to prefer to share it with stronger social ties and not weaker ones. In the case of collective action, the information may be a person’s dissatisfaction with a regime or her willingness to participate in a protest (Chwe 2000; Centola 2013). Given the sensitivity of this information, especially in oppressive regimes, a person may only be willing to pass it to her most trusted social ties. In the case of technology adoption, especially in the developing world, relevant information may be news of a development organization offering startup loans or handing out new technology like fertilizer. A person may judge the opportunity to be finite or selectively beneficial and prefer to share information of it with only her intimate social ties: kin or members of her salient in-group like her tribe (Larson and Lewis 2017). Crucially, in social networks, a person can choose whether to share information or whether to withhold it on a tie-by-tie basis.

In this conceptualization of information diffusion, a person in a social network will only spread information to a particular network neighbor if she (1) is presented with an opportunity to do so, and (2) is willing to share the information with that neighbor.

I account for these two features in a model in which a person has a finite number of opportunities to spread information to network neighbors. Individuals in the network have a type, which could represent ethnicity, tribe, political party, or salient social division. Ties between individuals of the same type are assumed to be strong, and ties connecting individuals with different types are assumed to be weak. Given an opportunity, a person always shares information with a same-type neighbor (strong tie) but occasionally withholds information from a different-type neighbor (weak tie).

I begin by demonstrating that the greater the hesitation to share with a weak tie, the greater is the reduction in diffusion. This result is straightforward, and leaves open the possibility that weak ties are at least better than nothing. I further show that in some cases, the presence of weak ties is strictly worse than having no ties at all in their place. The intuition is that, given a limited number of encounters, since weak ties transmit information at a lower rate than strong ties, the presence of weak ties can crowd out the use of stronger ties that are more conducive to diffusion.

I demonstrate these results by simulating diffusion on both hypothetical networks generated to possess properties of interest, as well as on two real social networks measured among Ugandan villagers (from Larson and Lewis 2017). These networks were shown to be sets of interpersonal relationships that can serve to pass information in in-person exchanges.

An implication of the present approach is that not all ties that could be added to a network are beneficial for information diffusion. Although the virtue of rewiring or adding random ties has been widely demonstrated (Newman 2000; Kleinberg 2002; Valente and Davis 1999; Pastor-Satorras and Vespignani 2001; Newman 2002; López-Pintado 2008), this article demonstrates that the presumption of full-capacity ties and perfect opportunity to transmit which underlies earlier approaches is necessary for random ties to be beneficial. By accounting for limited opportunities and varying capacity, the findings here help qualify these results. The findings presented here are consistent with those revealing that network modularity can improve information dissemination via social reinforcement (Centola 2010; Nematzadeh et al. 2014). The results here suggest that modularity is helpful for another reason: insofar as modularity is indicative of strong ties within the communities and weak ties across them, the presence of too many weak ties spanning communities can inhibit information spread *within* the communities as well.

## An opportunity model of information diffusion

Consider a social network *g* with a finite number of nodes. Suppose every node *i* has one of *n* types *τ*_*i*_∈{*τ* ^1^,…,*τ* ^*n*^}. A type is a descriptive feature of a node and is used to separate an in-group from out-groups, like membership in a certain tribe, political party, or salient social circle. Call a network *homogeneous* if *n*=1; that is, if all nodes have the same type. A network is *heterogeneous* if *n*>1.

A link, or “tie”, between nodes *i* and *j* in network *g* has a capacity *p*_*ij*_=*p*_*ji*_∈[0,1] such that when *i* (*j*) has information and encounters *j* (*i*), *j* (*i*) receives the information with probability *p*_*ij*_.

Let the capacity of a link be a function of the types of the nodes it connects. Specifically, let *p*_*ij*_>*p*_*kl*_ when *τ*_*i*_=*τ*_*j*_ and *τ*_*k*_≠*τ*_*l*_. Links have higher capacity when they connect nodes of the same type. This could be because a person trusts someone, is more interested in the wellbeing of someone, or is better able to communicate with someone when the two share a type. Call links between nodes of the same type “strong” and links between nodes of different types “weak”.

Now consider a simple model of information diffusion over time in which individuals may pass along new information to some network neighbors when presented with the opportunity. Call *i*’s neighbors in *g* *N*_*i*_(*g*). For simplicity, assume that all strong links have capacity *p*_*strong*_ and all weak links have capacity *p*_*weak*_. The diffusion process proceeds as follows:

- **t = 0** One node *i* is randomly selected and endowed with information.
- **t = 1** Seed *i* randomly encounters *x* of her network neighbors, *N*_*i*_(*g*). In each encounter, she passes information to the neighbor with probability *p*_*strong*_ if she and the neighbor are both the same type, and probability *p*_*weak*_<*p*_*strong*_ if they are different types.
- **t = 2** All *j* who learned information in *t*=1 randomly encounter *x* of their neighbors, *N*_*j*_(*g*), passing information with probabilities *p*_*strong*_ and *p*_*weak*_.
- **...** Repeats for all who learned information in the previous period until the information has reached everyone in the network or the spread halts.

In the model, an individual’s willingness to share information depends on the strength of a tie: she is more willing to share information along a strong tie (i.e. with same-type nodes) than along a weak tie (i.e. with different-type nodes). Her opportunity to share is determined by *x*. That *x* is fixed and not set to *#* *N*_*i*_(*g*) captures the realistic feature that although people may have many people whom they consider social contacts, and with whom they might share information if they had the chance, time is finite and so opportunities may be limited. Links in a social network do not guarantee the opportunity to spread information along them at any one point in time.^1^

These features differentiate this model from existing ones. Standard approaches to modeling diffusion on a network can be grouped into two main categories: threshold models (Granovetter 1978; Schelling 1978) (and variants (Berger 2001; Macy 1991; Macy and Willer 2002; Morris 2000; Valente 1996; Watts 2002; Young 2006)) and cascade models (Goldenberg et al. 2001a,b) (and variants (Carnes et al. 2007; Bharathi et al. 2007; Kempe et al. 2003; Kostka et al. 2008)). In the basic threshold model, each node has an exogenously-determined threshold which indicates the (possibly-weighted) proportion of her neighbors which must have heard the information in order for her to hear it too. The diffusion process typically unfolds by a few nodes selected at random to first receive the news, then in each subsequent period, all nodes whose thresholds are met become informed too.

In the basic independent cascade model, each node who recently learned information has a single chance to inform each neighbor, which succeeds with some exogenously-determined probability. These models proceed by endowing one or a few randomly chosen nodes with information, who inform each of their neighbors independently according to the exogenous success probabilities; those who receive information then inform each of their neighbors independently according to the success probabilities, and so on. Variants have largely focused on the dynamics of competing cascades, capturing the relative success of different rumors simultaneously spreading (Bharathi et al. 2007; Kostka et al. 2008).

The present approach is most similar to an independent cascade model, modified in two ways. First, here nodes have an exogenously given type, and the probability of successful transmission is a function of whether the types of the linked nodes match. Doing so builds a natural difference between weak and strong ties into the independent cascade model. Second, here nodes only have the chance to influence a possibly strict subset of their neighbors. The maximum number of neighbors that each node will encounter and possibly inform is an exogenous parameter, and captures the notion that a tie in a social network does not guarantee an opportunity to pass information to the tied node for any single piece of information.

### The downside to weak ties

I begin by demonstrating that the reduced capacity of weak ties inhibits diffusion throughout a community. To demonstrate the point, I simulate the spread of information via the process described above on two real social networks measured in rural Ugandan villages (from Larson and Lewis 2017). Nodes are villagers, links are measured social ties among the villagers, and node type is the villager’s ethnic group. In one of the two villages, Abalang, 94% of the 216 nodes belong to the same ethnic group, and 93% of the 660 links present in the network are between co-ethnics. In the other, Mugana, the largest ethnic group comprises only 62% of the 234 nodes, and 61% of the 965 links present in the network are among co-ethnics. Taking links between non-coethnics to be weak ties, 6% of ties in Abalang are weak, compared to 39% in Mugana.

**Fig. 1:**
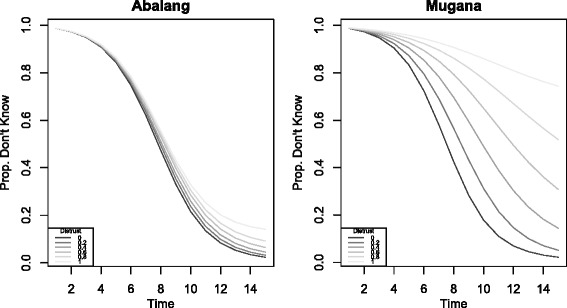
Proportion of network that remains uninformed by each timestep in simulated information spread on two real social networks, taking ethnic group as the relevant type. Simulation parameters set to *x*=2, *p*_*strong*_=1, and *p*_*weak*_={0,.2,.4,.6,.8,1}. Legend displays Distrust =1−*p*_*weak*_. The lower the capacity of weak ties (i.e. the greater the distrust), the more slowly information spreads throughout the whole community

Figure 1 shows the results of the simulated information spread. Each plot displays the proportion of the network that has not yet received the information by each timestep for different values of *p*_*weak*_. As the capacity of weak ties decreases, the likelihood that a node withholds information from her other-type neighbors increases, and this recurrence throughout the network dramatically slows the spread of information in the network with many weak ties. In the extreme case where nodes never share with different-type nodes, almost 80% of Mugana’s network is still uninformed by the 14th timestep, when, if these ties had the same capacity as strong ties, approximately no one would be uninformed. Even when the capacity of weak ties is non-zero, the rate of diffusion is substantially reduced in Mugana. While naturally, since Abalang has few weak ties, reducing their capacity does little to slow information diffusion there, in Mugana where weak ties are prevalent, even small reductions in capacity have large consequences for the reach of information.

The lower the capacity of weak ties, the worse information diffusion would be in networks with many weak ties. While this does suggest that accounting for tie capacity may be important for correctly predicting the rate and extent of information spread, it could still be case that the presence of weak ties is better than their absence. In the next section, I explore the consequences of adding new ties to networks, and show that there are plausible conditions under which the addition of a weak tie makes information diffusion strictly slower.

## Consequences of added links

The information diffusion process stipulated above has implications for links that are added to a network, which can affect overall diffusion. First, consider the consequences of a link randomly added to a network. Existing work consistently finds that randomly added or rewired ties improve information diffusion in homogeneous networks because random ties allow information to “jump” to distant network locations (Newman 2000; Kleinberg 2002). However, the diffusion process specified above introduces a second, potentially-competing effect in heterogeneous networks (and hence networks that may contain weak ties). Randomly added ties can change the composition of nodes’ neighborhoods. If neighborhoods are comprised of more ties to other-type nodes after the addition of ties, the expected number of neighbors who receive the information declines.

**Dual effects of random ties in heterogeneous networks** In networks comprised of nodes of varying types, the addition of a random tie can have two effects.

**Jump effect:** random ties allow information to jump across distant network locations, improving information dissemination. **Composition effect:** random ties change the composition of a node’s neighborhood, potentially impeding information dissemination.

In a heterogeneous network, which effect dominates– the jump effect which improves dissemination or the composition effect which hinders dissemination– depends on the relationship between homophily and the distribution of types in the network.

Node *i*’s network neighborhood *N*_*i*_(*g*) can be decomposed into $N_{i}^{same}(g)$, the subset of his network neighbors that are the same type as *i*, and $N_{i}^{dif}(g)$, the subset that are different. The expected number of nodes who receive information from *i* can then be written

1 $$\begin{array}{@{}rcl@{}} \frac{x}{\#N_{i}(g)} \left(\#N_{i}^{same}(g)p_{strong} + \#N_{i}^{dif}(g)p_{weak}\right), \end{array} $$

where *#* indicates the cardinality of a set.

The consequences of an additional tie added at random will depend on the proportion of the nodes in *g* that are the same type as *i*. Call $q^{\tau ^{k}}$ the proportion of nodes in *g* that are type *τ* ^*k*^. For simplicity, from any node *i*’s perspective, call $q_{i}^{same}$ the proportion of nodes of *i*’s type in *g*. Now a random link added to *N*_*i*_(*g*) will, on average, reduce the value of (1) whenever

2 $$\begin{array}{@{}rcl@{}} \frac{\#N_{i}^{same}(g)}{\#N_{i}(g)} - q_{i}^{same} > 0. \end{array} $$

That is, when the network is homophilous with respect to type so that a larger proportion of a node’s neighbors are his same type relative to the frequency of his type in the overall network, the addition of random ties will strictly reduce the expected number of people that that node informs.

The extent to which the expected number of nodes who receive information from *i* declines depends on the magnitude of the left hand side of (2). The greater the type homophily, the bigger the impact that random ties will have on reducing the expected number of people that a node informs.

Put another way, when new ties are likely to be weak, adding them to nodes with strong ties can reduce the expected number of people to whom those nodes pass information in their limited opportunities to do so. And of course, if ties are not added at random but we instead consider adding only weak ties (ties between people of different types), the reduction in the expected number of people informed by a node grows.

When this relationship is prevalent enough throughout a network, network-wide information dissemination can be strictly impeded by the addition of random ties, and by the addition of weak ties in particular. The next section demonstrates the aggregate results by simulating the above information diffusion process on hypothetical networks generated to isolate the relevant properties of interest.

## Simulated information spread

In this section I simulate the information diffusion process presented above on simple networks generated with varying levels of homophily and random tie additions.

### The downside to randomly added links

I begin by generating four hypothetical heterogeneous networks, each with two types of nodes. The networks have 234 nodes, half of which are each type, and 864 links. The numbers of nodes and links are the same as in the observed Mugana network described above. Each network is generated by randomly adding links according to a specified probability of attaching to a same-type node. One network is generated for each same-type node probability {.5,.65,.8,.95}. Let the difference between the proportion of same-type links present and the proportion of same-type links that would be observed by uniformly random link formation be called the network’s “homophily.” With two groups of equal size, the expected proportion of random same-type links is.5, yielding networks with homophily values {0,.15,.3,.45}.

I consider the consequences of increases in density for information diffusion by randomly adding links to the network. For each value of homophily, I add links such that the total number of links increases by a factor of 1, 2, 3, and 10.

Table 1 summarizes the interpretation of the model parameters and the values to which they are set in the simulations reported below.

**Table 1:** Model parameters

Parameter	Definition	Set to
*x*	Number of network neighbors a newly-informed node encounters in a period	2
*p*_*strong*_	Probability pass news to an encountered neighbor if neighbor is same type	1
*p*_*weak*_	Probability pass news to an encountered neighbor if neighbor is different type	{.25,.5}
*τ*={*τ* ^1^,…,*τ* ^*n*^}	Set of types	{*τ* ^1^,*τ* ^2^},
$q^{\tau ^{k}}$	Proportion of type *τ* ^*k*^∈*τ*={*τ* ^1^,…,*τ* ^*n*^} present in the network	$\frac {1}{2}$
Homophily	Prop. same-type ties in network minus prop. same-type ties expected under random tie formation	{0,.15,.3,.45}
Diversity	Number of types, or “groups,” present in network	2

**Fig. 2:**
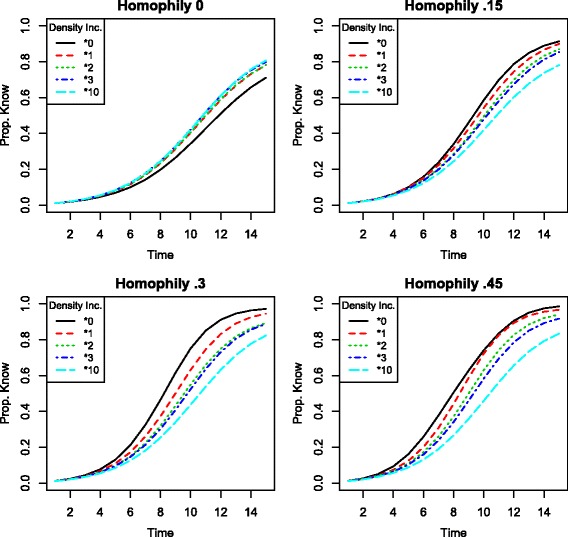
Proportion of network informed by each timestep in simulated information spread on a network with *τ*={*τ* ^1^,*τ* ^2^}, and $q^{\tau ^{1}} = q^{\tau ^{2}} = \frac {1}{2}$. Simulation parameters set to *x*=2, *p*_*strong*_=1, and *p*_*weak*_=.5. When homophily = 0, random ties will not change neighborhood compositions on average, so the jump effect dominates and increasing density strictly improves information diffusion. At greater values of homophily, increasing density does change neighborhood compositions and strictly impedes information diffusion

Figure 2 shows the results of the simulated information diffusion process on each of these networks, grouped by homophily value. In each quadrant, the curves plot the average proportion of the network that is informed by the timestep on the horizontal axis over a set of 500 simulations for a particular value of density increase. Since the population is finite, *p*_*strong*_>0, and *p*_*weak*_>0, diffusion follows the characteristic s-shape. The lower the curve, the slower the diffusion.^2^

When the network exhibits no homophily (top left), randomly adding links can improve information dissemination. In this case, since the composition of the population matches the composition of neighborhoods on average, randomly adding links has no composition effect. In expectation, neighborhoods retain the same proportion of weak ties. The jump effect dominates, improving information dissemination on net.

When network neighborhoods contain more same-type links than would be expected based on the overall network composition (exhibit positive homophily), the composition effect is present alongside the jump effect. In the cases of positive homophily shown in Fig. 2, the composition effect dominates: an increase in density actually impedes information diffusion. Additional ties result in neighborhoods with more weak ties than before the addition. The greater the number of links added, the worse the diffusion.

Note that the number of randomly-added ties is large in these simulations, in some cases increasing the number of links in the network many-fold. Under standard epidemiological models of information diffusion, the improvement in diffusion would be vast. Here, these large additions actually *reduce* the spread of information. Moreover, these simulations assume that individuals share with other types half of the time (*p*_*dif*_=.5). When people are more hesitant to share with other types so that *p*_*weak*_ is smaller, the reduction in information spread is even greater.

**Fig. 3:**
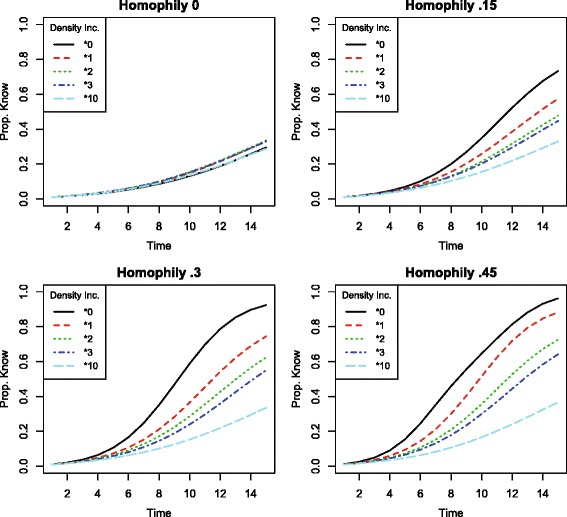
Proportion of network informed by each timestep in simulated information spread on a network with *τ*={*τ* ^1^,*τ* ^2^}, and $q^{\tau ^{1}} = q^{\tau ^{2}} = \frac {1}{2}$. Simulation parameters set to *x*=2, *p*_*strong*_=1, and *p*_*weak*_=.25. When homophily is positive, increasing density changes neighborhood compositions and strictly impedes information diffusion; at this lower value of *p*_*weak*_, the impediment is even greater

Figure 3 reproduces the simulations from Fig. 2 with lower-capacity weak ties. Here *p*_*weak*_ is set to.25. Comparing the two figures makes clear that when a person is less willing to share along weak ties, the addition of ties, many of which will be weak, has an even stronger negative effect on diffusion.

## Adding weak ties to Mugana

**Fig. 4:**
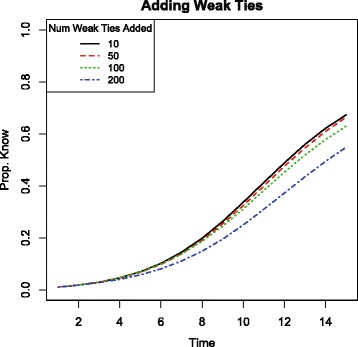
Proportion of network informed by each timestep in simulated information spread in Mugana with new weak ties added to the network. Simulation parameters set to *x*=2, *p*_*same*_=1, and *p*_*weak*_=.4. Adding weak ties makes diffusion strictly slower

The last section showed that adding ties at random to a network can impede information diffusion. The impediment is due to the additional ties that are weak and so are lower capacity. Now I show that adding only weak ties– only ties that connect nodes with different types– would impede information diffusion in the real network from Mugana. Mugana has nodes with different types (ethnicities), so there are many possible weak links that can be added. Figure 4 compares the rate of diffusion for different numbers of weak ties added at random to the network.

Adding a larger and larger number of weak ties to the existing network increases the total number of links present, but actually reduces the rate of information spread throughout the village. The reason is that although the weak ties added at random are allowing information to possibly jump to new regions of the network (to non-coethnics), nodes with strong ties who received new weak ties have changed neighborhoods. That change results in a different option set for spreading information. These nodes, like all nodes, will encounter *x* of their neighbors. The more that the neighborhood is comprised of non-coethnics, the more likely these opportunities will include those to whom information does not flow as freely. Adding weak ties effectively crowds out the use of the strong ties which transmit information more readily.

**Fig. 5:**
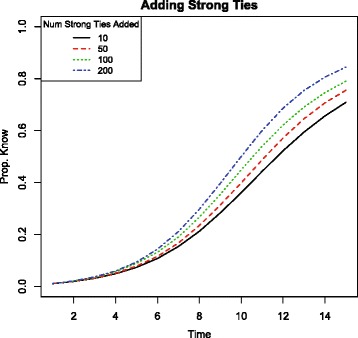
Proportion of network informed by each timestep in simulated information spread in Mugana with new *strong* ties added to the network. Simulation parameters set to *x*=2, *p*_*same*_=1, and *p*_*weak*_=.4. In contrast to the addition of weak ties in Fig. 4, adding strong ties improves diffusion

For reference, Fig. 5 shows the results of the equivalent simulations in which strong instead of weak ties are added. In contrast to the consequences of adding weak ties shown in Fig. 4, increasing density by adding only strong ties– ties that connect coethnics– strictly *improves* diffusion.

### Discussion

The downside of weak ties depends on two features of the information spread: the limited number of opportunities to share a piece of information (x) and the lower capacity of the weak ties (*p*_*weak*_<*p*_*strong*_). As *p*_*weak*_ and *x* grow, the downside becomes less severe, and can be overwhelmed by the jump effect.

In the simulations, the number of opportunities to share, *x*, is set to 2. When *x* is larger, information dissemination occurs more rapidly. Whether weak ties hinder the process of information dissemination relative to their absence depends on the size of *x* relative to the number of social contacts nodes have in the network.^3^ In the case of Mugana, on average, people have just over 7 social ties. The above simulation then assumes that people encounter under a third of their complete set of social contacts when they have a piece of news to share. In the original study of Abalang and Mugana, surveys revealed that villagers in fact had the opportunity to share a new piece of news with approximately one third of their social contacts on average (Larson and Lewis 2017, footnote 18).

Just how constrained a person’s opportunities to share information are– the true value of *x*– surely depends on the context being studied. Two features of the context that likely bear on opportunities to share are the type of information that can be passed along, and the environment in which people interact and possibly share information with others. For instance, information that is potentially sensitive, like news of an indiscretion or revelations of a desire to protest against an oppressive regime, may only be passed in private, face-to-face encounters with others. Even if a person finds herself in a large group with all of her social contacts present, she may refrain from broadcasting the news there, instead waiting for private encounters to more delicately share the information. Since private encounters themselves take time, and finding times when others are not present may be difficult, a person may find herself with a low *x*– few opportunities to share relative to the size of her whole set of social contacts.

For information that is less sensitive, certain environments may be more conducive to plentiful opportunities to share it than others. If communities regularly hold town hall meetings, or organize into large gatherings that include many social contacts at once, people may be able to share news of the day with a larger number of their social contacts (and may feel free to do so if the news is not sensitive). To capture information diffusion throughout such a group, *x* could be set higher. On the other hand, in an environment of very busy people who are engaged in tasks that keep them away from their social contacts for long stretches– people who farm or mine for long stretches at a time, say– small values of *x* relative to neighborhood size may reasonably capture the limited opportunities to pass information along.

Note that constraining the number of opportunities to be less than a node’s neighborhood size drives the net downside to weak ties. If everyone encountered every one of their social contacts when they had new information, the presence of weak ties would not have a net negative effect on diffusion. Weak ties would be less effective channels through which information spreads; their presence would not be harmful, but would simply not add as much to the speed and reach of information as strong ties. If, however, a person’s opportunities are constrained to be less than her full set of social contacts, then the presence of weak ties can have a net negative effect. Just how small *x* is relative to a person’s total number of social contacts determines just how damaging the presence of weak ties is.

## Conclusion

The common approach to the study of information diffusion assumes that any tie is better than no tie. So long as weak ties can transmit some information, their presence should be favorable to diffusion, and since their presence tends to connect people of different types (in different subgroups or communities), weak ties should be especially conducive to the rapid diffusion of information throughout a group.

However, this intuition relies on models of diffusion imported from fields such as epidemiology which fail to capture two features of real groups of people deciding whether or not to share information with ties in their social network. First, people may not have the opportunity to share information with every social tie they have at any particular moment. Links in a social network indicate a relationship through which information *could* travel, but the existence of a social relationship by no means guarantees that one person will certainly and immediately share any information she has with the other. Second, even given the opportunity, a person may prefer to withhold information rather than share it, and she may do so selectively based on the potential recipient. This preference may arise because she does not trust the recipient, does not want to share the benefit the information offers with the recipient, or may be less able to make the recipient understand.

Accounting for these features reveals that, while the well-understood “jump effect” can in fact be helpful, a second “composition effect” can result in random ties impeding the spread of information, even on net. These dual effects call into question the logic that a tie with non-zero capacity must be better than no tie at all. In fact, weak ties may actually reduce diffusion on net, even relative to a network in which no ties were present in their place. Moreover, the common finding that the addition of random ties to a network will improve diffusion overall may not hold for heterogeneous social networks. In heterogeneous groups, especially ones with high homophily, greater density can actually strictly reduce the speed with which information spreads throughout a network.

To demonstrate these points, I use real networks and node types to show that the lower the capacity of weak ties, the worse is information diffusion. Next, I isolate the features of networks that matter for weak ties helping or hurting, generate hypothetical networks that vary in these properties, and demonstrate that the addition of weak ties can have a net negative effect on overall diffusion. Finally I return to the real networks and show that adding weak ties there could strictly reduce the speed and reach of new information flowing through the social network.

In addition to the implications for information dissemination in general, these results also speak to the broad study of diversity. If the presence of more groups in an area makes ties more likely to be weak, then information diffusion may be impeded and the addition of new weak ties may be especially unhelpful (see Larson 2016). This logic can be informative for the so-called “curse of diversity” observed in the developing world (Miguel and Gugerty 2005; Larson 2017). Beyond an explanation for why diversity may hinder the spread of information, this framework also suggests a reason why “cross-cutting cleavages”, or membership in overlapping groups, may be particularly beneficial (Lipset and Rokkan 1967). If the capacity of a tie is higher when it connects two people who share membership in at least one group, then information may spread better in communities featuring a large extent of shared membership compared to those featuring stark separation between groups.

These results also highlight important considerations for future research. The argument for why weak ties may have a lower capacity pertains to situations in which a node holds information, and the onus is on her to share it or not. This is most likely to be the case when the information is novel. In such a supply-driven situation, potential recipients do not know they do not know something. Contrast this with a demand-driven situation such as the case of a job-seeker looking for tips about employment opportunities. In this case, the uninformed node– the person looking for a job– knows there is information she does not have. Because she knows there may be relevant information available, she may even more actively seek it out from her weak ties. In this situation, the Granovetter logic is likely to hold: if she actively approaches her weak ties, she may access information from far away in the network that she otherwise would have missed.

The situation of truly novel information is likely to be supply-driven. Consider an international non-governmental organization that arrives in a community and tells a few people about an opportunity to participate in a new, potentially-profitable program, or a brand new social movement or rebel group that begins to mobilize in an area. People who are not initially informed about the program or the movements are also likely unaware that they are missing information about a program or movements. They do not know to seek out information; instead, it is those who possess the information who are tasked with sharing it or not. If weak ties are lower capacity and people have constrained opportunities to share the information with others, then the Granovetter logic may no longer hold.

In demand-driven information environments, the fact that a node may specifically approach her weak ties, and the fact that she may do so with greater persistence, may overcome the problems of limited capacity and render weak ties beneficial. This article shows that in supply-driven environments, if opportunities are constrained and people have reason to selectively share information with some instead of others, then weak ties may at best be weak for the diffusion of novel information.

Furthermore, the findings here hinge on limited opportunities to share– on a value of *x* that is less than a person’s full set of social contacts. While this is very likely to be a constraint in face-to-face interactions, communications technology may relax this constraint in some settings and for some information types. Pinning down exactly when weak ties are likely to have a net negative effect based on considerations like these will be an important next step for the theoretical and empirical study of information diffusion.

## Endnotes

^1^ For simplicity, the diffusion process assumes that a node participates in spreading information in one window of time, and then never tries to spread it again. This assumption is shared by independent cascade models (Goldenberg et al. 2001a,b). While the results are starkest under this assumption, they continue to hold under a weaker assumption. Even if a node is presented with the opportunity to share information with a randomly chosen set of *x* of her contacts in one timestep, and then with an independently drawn set of *x* of her contacts in the next timestep, and so on over time, the comparisons below will still hold. This modification would dramatically speed the diffusion process, but the comparison between networks with weak ties present and those with weak ties absent would still hold: constrained opportunities admit the possibility that weak ties will impede the process.

^2^ This represents an impediment to diffusion in the sense that information reaches people more slowly, and also in the sense that by any given point in time, fewer people are informed.

^3^ For any node, if *x* is smaller than the size of that node’s neighborhood, weak ties incident to that node can crowd out strong ties. The net effect on diffusion depends on whether the jump effect is overwhelmed by the composition effect; see Section “Consequences of added links”.
